# Effects of Wheatgrass Juice Supplementation in Common Carp (*Cyprinus carpio*): Growth Performance, Biochemical Profile, Oxidative Status, and Cultivable Intestinal Bacteria

**DOI:** 10.3390/life16101619

**Published:** 2026-09-27

**Authors:** Bălbărau Adrian, Cristian-Alin Barbacariu, Lenuța Dîrvariu, Dana Andreea Șerban, Marian Burducea, Gabriela Dumitru, Elena Todirașcu-Ciornea, Rîmbu Cristina Mihaela, Acatrinei Dumitru, Miron Liviu

**Affiliations:** 1 Faculty of Veterinary Medicine, “Ion Ionescu de la Brad” Iași University of Life Sciences, Aleea Mihail Sadoveanu nr. 8, 700489 Iasi, Romania; balbarau.adrian@yahoo.com (B.A.);; 2 Research and Development Station for Aquaculture and Aquatic Ecology, “Alexandru Ioan Cuza” University, Carol I, 20A, 700505 Iasi, Romaniadana.serban@uaic.ro (D.A.Ș.);; 3 Faculty of Biology, “Alexandru Ioan Cuza” University, Carol I, 20A, 700505 Iasi, Romania

**Keywords:** carp fingerlings (*Cyprinus carpio*), wheatgrass juice, biochemical blood parameters, bacteriological, oxidative stress, clinical health

## Abstract

Aquaculture faces numerous challenges related to fish health. To explore new solutions, the objective of this study was to test the effects of wheatgrass juice (WGJ) as feed supplement on growth performance, blood biochemical profile, bacteriological balance, and oxidative stress in common carp (*Cyprinus carpio*). Carp fingerlings (8.8 g initial body weight) were fed diets containing 4% or 8% WGJ in a recirculating aquaculture system for eight weeks. Most growth performance parameters did not differ significantly among dietary treatments, although the viscerosomatic index (VSI) was significantly lower in WGJ-supplemented groups. WGJ supplementation was associated with changes in oxidative-status parameters, including increased antioxidant enzyme activities and reduced malondialdehyde levels, particularly at the 8% inclusion level. Blood biochemical parameters showed no evidence of adverse effects on liver or kidney function. Total protein, globulin, and albumin did not differ significantly among treatments. Microbiological examination highlighted a decrease in pathogenic bacteria, including sulfite-reducing clostridia, with a 4% WGJ diet showing the most pronounced inhibitory effects. These results indicate that WGJ supplementation may influence selected biochemical, oxidative-status, and cultivable intestinal bacterial parameters in common carp. Further studies are required to determine the underlying mechanisms and the practical relevance of these effects.

## 1. Introduction

Common carp (*Cyprinus carpio*) is cultivated worldwide, accounting for 7.7% of global fish production in 2018 [1]. In Romania, this species represents the most extensively farmed fish, with an estimated production of 4365.45 tons in 2018, constituting 35% of the country’s total fish output [2].

To meet the growing demand, aquaculture farmers often increase the density of fish stocks. However, high stocking densities are associated with oxidative stress, reduced immune function, and the onset of various diseases, ultimately leading to diminished production [3]. Diseases are a big problem in intensive aquaculture systems, with global reports indicating that millions of tons of fish are lost annually to disease outbreaks, leading to an important economic impact [4]. In Romania, fish are commonly affected by bacterial agents such as *Aeromonas*, *Vibrio*, and *Pseudomonas* [5].

Antibiotics are frequently employed as prophylactic measures in aquaculture [6]. Due to the limited availability of antibiotics specifically designed for this sector, medications intended for other areas of veterinary medicine are often used. This practice, coupled with the overuse of certain antibiotics, has led to the emergence of antimicrobial-resistant bacterial strains, posing risks to both fish and human health [6,7,8]. Previous studies indicate that bacterial strains in aquaculture have developed resistance to at least five antibacterial agents, including ampicillin, chloramphenicol, flumequine, furazolidone, and trimethoprim–sulfamethoxazole [9]. This being said, the aquaculture sector is in need of alternatives to conventional synthetic drugs that do not pose a risk of promoting antimicrobial resistance and can be safely used in aquatic environments.

Aquatic environments also provide optimal conditions for the development, persistence, and evolution of parasitic organisms. Infections are ubiquitous among fish, imposing significant fitness costs and contributing to the array of environmental stressors that negatively impact their health [10,11,12]. Diseases caused by parasites are typically managed using chemical treatments such as formalin, sodium chloride, acetic acid, potassium permanganate, copper sulfate, praziquantel, and emamectin benzoate. However, these approaches can select for resistant parasites, harm the environment, and pose risks to human health through the accumulation of chemical residues in farmed fish [13,14,15,16].

As an alternative, plant-derived dietary supplements have emerged as a safer prophylactic and disease-control strategy in aquaculture [17]. Phytogenic additives, derived from plants, are increasingly utilized due to their diverse benefits, including enhanced growth, reduced oxidative stress, strengthened immune responses, and minimal environmental impact. These properties make phytogenic additives a viable alternative to synthetic drugs for managing fish health [18].

Wheatgrass juice is an extract obtained from 10–15-day-old *Triticum aestivum* plants and has been recognized for its therapeutic properties, including antioxidant, cardioprotective, blood sugar-regulating, and immune-stimulating effects [19,20,21,22]. It contains a rich array of vitamins, minerals, enzymes, and amino acids [23,24,25,26].

In addition, wheatgrass juice is abundant in vitamin K, sulfur, and chlorophyll, which are reported to combat infections, promote wound healing, reduce inflammatory processes, and enhance immune function. These components may also reduce the risk of parasitic infestations by inhibiting the metabolic activation of carcinogens and increasing the immune response of the fish [27,28]. All the findings mentioned above supported by previous studies were very rarely linked to the applicability of wheatgrass juice administration in fish and aquatic environments.

Although previous studies have reported beneficial effects of wheatgrass juice on growth performance, blood biochemical parameters, and oxidative status in common carp [18,29], its effects on oxidative responses across different tissues and on selected cultivable intestinal bacterial groups remain insufficiently characterized. In particular, it is unclear whether different dietary inclusion levels produce similar or concentration-dependent responses. The aim of the present study was to test the effects of 4% and 8% WGJ supplementation on growth performance, blood biochemical parameters, body composition, oxidative status in muscle, liver, and intestine and selected cultivable intestinal bacterial indicators in common carp.

## 2. Materials and Methods

### 2.1. Fish Trial

The experiment complied with Romanian animal welfare legislation and Directive 2010/63/EU. Ethical approval was granted by the Faculty of Biology Ethics Committee “Alexandru Ioan Cuza” University of Iași, Romania. (No. 377/29.01.2024).Common carp (*Cyprinus carpio* L.; initial body weight: 8.8 g) were maintained in an indoor recirculating aquaculture system (RAS). This size was selected as carp fingerlings are highly vulnerable to environmental stressors, and their growth rate is significantly accelerated during this stage. Enhancing cultivation technology, particularly through the use of health-promoting feeds, directly contributes to the productivity and sustainability of carp aquaculture systems. Each experimental variant was conducted in triplicate, with 30 fish per tank. The trial was conducted between 13th of October 2025 and 18th of February 2026.

The RAS included 0.75 m^3^ fiberglass tanks, mechanical and biological filtration, UV treatment, water circulation pumps, and sensors for continuous water-quality monitoring. A 10 h daily photoperiod was provided using natural and fluorescent light, and salinity was maintained at 0.1 mg/L. The tank was considered the experimental unit because dietary treatments were assigned at the tank level. Three independent replicate tanks were used for each dietary treatment, with 30 fish maintained in each tank. Accordingly, the effective statistical sample size for treatment comparisons was three tanks per dietary treatment (*n* = 3). When measurements were obtained from multiple fish within the same tank, individual-fish measurements were averaged within tank before statistical analysis so that each tank contributed one observation per endpoint. Technical replicate measurements obtained from the same biological sample were likewise averaged before statistical analysis and were not treated as independent observations. The design was also consistent with the experimental approach used in our previous study [18].

Outcome assessment and laboratory analyses were performed under blinded conditions.

Water parameters were closely monitored to ensure optimal environmental conditions. Temperature and dissolved oxygen (DO) were monitored with a Hach HQ30d portable oxygen meter (Hach Company, Loveland, CO, USA), whereas pH and conductivity were measured using a Hach HQ11d portable multiparameter device (Hach Company, Loveland, CO, USA). Ammonia, nitrite, nitrate, and phosphate concentrations were determined with a Hanna Iris HI801 Spectrophotometer and corresponding reagent kits (Hanna Instruments, Sălaj, Romania). Detailed water-quality data are presented in Table 1.

### 2.2. Wheatgrass Juice Extraction

Wheatgrass juice was prepared following the method described by Dumitru et al. (2018) [29]. Wheat seeds were germinated in the dark for three days in 1-L black plastic pots filled with forest soil collected from a region near Iași.

After germination, the plants were cultivated under natural light, with watering performed twice daily using 50 mL of water per pot. After 10 to 15 days, the plants reached the optimal harvest height of 15 cm. Harvesting was conducted by cutting the stalk 2 cm above the soil surface. The juice was then extracted using a cold-pressing method to preserve its nutritional and bioactive properties.

### 2.3. Experimental Diets

The experimental diets were prepared by grinding, mixing, extrusion, and pelletization processes, with a pellet size of 3 mm. The proximate composition of the feed was determined using a DA 7250 NIR Analyzer (Perten Instruments, Hägersten, Sweden). Three dietary variants were tested:

V0 (control): A diet with 0% wheatgrass juice (WGJ).

V1: A low-dose diet containing 4% WGJ.

V2: A high-dose diet containing 8% WGJ.

The dietary inclusion levels of WGJ (4% and 8%) were selected based on our previous study in common carp, which demonstrated the potential of wheatgrass juice as a dietary feed additive [18]. The present study was designed to further investigate the effects of these inclusion levels on growth performance, blood biochemical parameters, oxidative status, and selected cultivable intestinal bacterial groups [18].

Wheatgrass (*Triticum aestivum*) was harvested at approximately 10–15 days of growth and freshly processed daily by cold pressing. The resulting wheatgrass juice (WGJ) was applied to the experimental feed by fine spraying, followed by manual mixing to facilitate homogeneous distribution throughout the feed. The treated feed was subsequently allowed to dry at room temperature for approximately 30 min without direct exposure to sunlight before being provided to the fish.

Wheatgrass juice was incorporated into the experimental diets at 4% (V1) and 8% (V2), while the control diet contained no wheatgrass juice (V0). Feed was supplied at 15% of fish biomass per day, and the amount of WGJ applied was adjusted according to the daily feed quantity and treatment level. With 30 fish maintained in each tank, the final-body-weight-based daily feed quantities were approximately 120.1–127.5 g per tank, corresponding to approximately 4.80–5.10 mL WGJ for the 4% treatment and 9.61–10.20 mL WGJ for the 8% treatment.

The experimental diets were formulated to provide comparable crude protein levels among treatments. The analyzed crude protein content was approximately 30% across the dietary groups (V0, V1, and V2). However, because dietary energy was not determined, the diets are not considered demonstrably isoenergetic. Other proximate components showed relatively small differences among treatments, which may reflect the contribution of wheatgrass juice to the final diet composition. The ingredients and proximate composition of the experimental diets are presented in Table 2.

At the conclusion of the trial, the survival rate of the fish was 100%, indicating the suitability of the experimental conditions and diets.

### 2.4. Fish Growth Indices

Fish length and body weight were measured periodically (*n* = 20).

The growth indices measured were as follows:

- IBW (g) —initial body weight;
- FBW (g)—final body weight;
- WG (g)—weight gain = FBW − IBW;
- FCR—feed conversion ratio = Feed intake/WG;
- CF—condition factor = (FBW/L^3^) ∗ 100;
- PER—protein efficiency ratio = WG/protein intake;
- RGR—relative growth rate (g/g day^−1^) = WG/days of experiment/IBW;
- SGR—specific growth rate (% day^−1^) = (ln FBW − ln IBW)/days of experiment ×100;
- HSI (hepatosomatic index, %) = 100 × [final liver weight (g)/final body weight (g)];
- VSI (viscerosomatic index, %) = 100 × [final visceral weight (g)/final body weight (g)].

### 2.5. Proximate Body Composition

Proximate body composition parameters were determined using DA 7250 NIR Analyzer (Perten Instruments). Muscle samples from five fish per treatment were analyzed in triplicate, and the results are presented as means ± standard error.

### 2.6. Blood Parameters

The fish were anesthetized with clove oil (0.2%), and blood was collected by cardiac puncture. The samples were analyzed with the MNCHIP Pointcare V2 Analyzer Tianjin MNCHIP Technologies Co., Ltd., Tianjin, China.

The investigated parameters were: ALB—albumin, TP—total protein, GLO—globulin, A/G—albumin/globulin, Ca^2+^—calcium, GLU—glucose, BUN—blood urea nitrogen, P—phosphorus, AMY—amylase, CHOL—cholesterol, ALT—alanine aminotransferase, TBIL—total bilirubin, ALP—alkaline phosphatase, CRE—creatinine, BUN/CRE ratio and CK—creatine kinase.

### 2.7. Oxidative Stress

At the end of the trial, fish were euthanized with 2% clove oil, and liver, muscle, and intestinal samples were collected. Two fish per tank were analyzed, and their values were averaged for each parameter and tissue, with the tank considered the experimental unit (*n* = 3 per treatment). Tissues were homogenized (1:10, *w*/*v*) in ice-cold 0.1 M potassium phosphate buffer containing 1.15% KCl (pH 7.4), followed by centrifugation at 3000 rpm for 20 min at 4 °C. The resulting supernatants were used to determine SOD, CAT, GPX, and MDA according to Dumitru et al. (2019) [30]. Results were normalized to soluble protein content determined by the Bradford method [31].

### 2.8. Microbiological Examination

The intestinal microbiota is closely related to the physiological functions of an organism, playing an important role in the health of the host. Studies conducted on fish have shown that the quality of the microbiota influenced their nutritional metabolism, immunity, and physiological performance [32]. The complexity of the fish microbiota is directly influenced by diet. The composition of the diet determines the composition and diversity of the intestinal microbiota, as the nutrient substrates can promote the multiplication of certain microorganisms to the detriment of others, altering the structure of intestinal microbial communities [33]. Cultivable intestinal bacterial groups were evaluated in common carp (*Cyprinus carpio*) fry fed the control diet (V0) or diets supplemented with 4% (V1) and 8% WGJ (V2). The total numbers of anaerobic bacteria (TNAB), sulfite-reducing clostridia (TNS-RCS), and Enterobacteriaceae (TNE) were determined and expressed as CFU/g of intestinal content. Following necropsy, intestinal contents were aseptically collected with a Pasteur pipette. Culture procedures and media were specific to each microbial indicator, as described in [34].

#### 2.8.1. Determination of TNAB (Total Number of Anaerobic Bacteria)

Aerobic bacterial load was determined from decimal dilutions (10^−1^–10^−3^) prepared in physiological saline. Aliquots (1 mL) were plated with approximately 15 mL of Plate Count Agar (PCA, Ecolab, 1 Ecolab Place, St. Paul, MN 55102, USA) cooled to 45 °C and incubated aerobically at 25 °C for 48 h. Colonies were counted, corrected for the dilution factor, and expressed as CFU/g of intestinal content.

#### 2.8.2. Determination of TNE (Total Number of Enterobacteriaceae)

The working stages were the same as for TNBA/g; the differences consisted in the use of Bromocresol purple lactose Agar (BCP Biorad, 1000 Alfred Nobel Drive, Hercules, CA 94547, USA).

#### 2.8.3. Determination of TNS-RCS (Total Number of Sulfite-Reducing Clostridia)

Sulfite-reducing clostridia are strictly anaerobic germs that require anaerobic conditions, created by using certain agents and the addition of CO_2_-generating systems (bioMérieux, 376 Chemin de l’Orme, 69280 Marcy-l’Étoile, France). For testing, Sulfite–Polymyxin B–Sulfadiazine Agar (Difco Laboratories, 920 Henry St, Detroit, MI 48201, USA) was used, as it is an efficient medium recommended for the isolation and identification of sulfite-reducing clostridia.

### 2.9. Statistical Analysis

Statistical analyses were performed using SPSS Statistics 21 (IBM Corp., Armonk, NY, USA). Normality and homogeneity of variance were assessed using the Shapiro–Wilk and Levene tests, respectively, and both assumptions were met (*p* > 0.05). Differences among treatments were evaluated by one-way ANOVA followed by Tukey’s post hoc test. Statistical significance was set at *p* < 0.05, and results are presented as mean ± SE.

## 3. Results

### 3.1. Growth Parameters

Growth-performance data are presented in Table 3. Final body weight, weight gain, feed conversion ratio, condition factor, protein efficiency ratio, relative growth rate, specific growth rate, and hepatosomatic index did not differ significantly among treatments. The viscerosomatic index was significantly lower in V1 and V2 than in V0 (*p* = 0.019). Therefore, the numerical increase in weight gain and decrease in feed conversion ratio in the WGJ groups should be interpreted as non-significant trends rather than treatment effects.

### 3.2. Blood Parameters

The biochemical parameters of blood are shown in Table 4. No significant differences were observed among dietary treatments for any of the assessed blood biochemical parameters. Most parameters remained within the reported physiological ranges, although ALT activity exceeded the reference range in all experimental groups.

Two fish were sampled from each tank for blood biochemical analyses. Values from the two fish within each tank were averaged before statistical analysis; therefore, each tank contributed one observation, and the effective statistical sample size was three tanks per dietary treatment (*n* = 3).

### 3.3. Proximate Body Composition

Proximate body composition parameters are shown in Table 5.

Salt content differed significantly among treatments (*p* = 0.050), with lower values observed in V1 and V2 compared with the control. No significant differences were detected for fat, moisture, protein, or collagen content.

For comparison, proximate body composition values for common carp reported in the literature are presented in Table 6.

### 3.4. Oxidative Status

Figure 1 presents the oxidative status of carp after the feed trial with WGJ. In muscle tissue, the control group showed an average SOD activity of 2.244 ± 0.223 USOD/mg protein. In the group treated with 4% WGJ (V1), the enzyme activity increased slightly to 2.634 ± 0.154 USOD/mg protein. In the group treated with 8% WGJ (V2), the activity rose further to an average of 3.193 ± 0.255 USOD/mg protein. In liver tissue, SOD activity was higher in both experimental groups compared to the control. The control group recorded 3.928 ± 0.285 USOD/mg protein, while the V1 and V2 groups showed activity levels of 4.227 ± 0.253 USOD/mg protein and 4.633 ± 0.296 USOD/mg protein, respectively. In intestine tissue, no major differences were observed between the control group and the V1 group. However, statistical analysis revealed significant differences (*p* < 0.01) between the V1 and V2 groups.

Catalase (CAT), another crucial antioxidant enzyme, showed variable activity depending on the tissue and experimental group. In muscle tissue, CAT activity ranged from 21.697 ± 1.755 UCAT/mg protein in the V1 group to 34.582 ± 2.956 UCAT/mg protein in the V2 group. In liver tissue, CAT activity was highest in the V2 group (53.556 ± 1.655 UCAT/mg protein), followed by the V1 group (43.215 ± 1.985 UCAT/mg protein) and the control group (39.945 ± 1.794 UCAT/mg protein). In intestine tissue, CAT activity increased slightly in the experimental groups (17.293 ± 1.235 UCAT/mg protein for V1 and 18.215 ± 1.765 UCAT/mg protein for V2) compared to the control group (14.232 ± 1.535 UCAT/mg protein), though these differences were not statistically significant.

Glutathione Peroxidase (GPX), an intracellular antioxidant enzyme, also demonstrated increased activity in the experimental groups: In muscle tissue, GPX activity was 0.877 ± 0.115 UGPX/mg protein in the control group. It increased to 2.751 ± 0.175 UGPX/mg protein in the V1 group and 3.433 ± 0.193 UGPX/mg protein in the V2 group. In liver tissue, the control group showed the lowest GPX activity (3.582 ± 0.182 UGPX/mg protein), while the V1 and V2 groups exhibited higher activity levels (4.146 ± 0.095 UGPX/mg protein and 4.952 ± 0.196 UGPX/mg protein, respectively).

Malondialdehyde (MDA), an indicator of lipid peroxidation caused by oxidative stress, was measured across tissue types. In intestine tissue, the lowest MDA concentration was observed in the V2 group (1.083 ± 0.045 nM/mg protein), while the control group exhibited the highest concentration (1.465 ± 0.098 nM/mg protein). Similar trends were observed in muscle and liver tissues.

### 3.5. Intestinal Microbiological Evaluation

Figure 2 presents the evaluation of the selected microbial groups from the intestine of carp. From the graphical representation of the obtained results, it is evident that all microbiological indicators have undergone changes compared to the control group (V0), but the emphasis is placed on the group treated with diet V1. In this sample, the total number of anaerobic bacteria (TNBA) decreased from 10,173.33 CFU/g (V0 group) to 2620 CFU/g (V1), total number of Enterobacteriaceae (TNE) was reduced from 4720 CFU/g (V0—control group) to 2740 CFU/g (V1), and the sulfite-reducing clostridia (TNS-RCS) were completely inhibited from 46.66 CFU/g to 0 CFU/g.

Regarding the values of the indicators tested for the group of fish fed with diet V2, these are closer to the average values of the control group, except for the TNS-RCS indicator, which was inhibited (similar to the V1 group). For the TNBA indicator, there is a slight increase (10,633.33 CFU/g) above the value of the control group (10,173.33 CFU/g). Additionally, small changes were also identified in the TNE indicator, with the values obtained for V2 being slightly lower (3846.70 CFU/g) compared to the control group values (4720.00 CFU/g).

## 4. Discussion

WGJ supplementation did not significantly affect final body weight, weight gain, feed conversion ratio, protein efficiency ratio, relative growth rate, specific growth rate, or hepatosomatic index. Although weight gain increased and feed conversion ratio decreased numerically in the supplemented groups, these differences were not statistically significant and therefore do not demonstrate improved production performance. The lower viscerosomatic index in both WGJ groups indicates a change in the relative mass of the viscera, but the present measurements do not identify the tissue or mechanism responsible. This finding should therefore be interpreted cautiously and examined together with organ-specific and histological measurements in future work. The absence of a significant growth response may reflect the eight-week exposure period, the tested inclusion levels, diet composition, or the limited number of independent tank replicates. Previous work in common carp has shown that responses to wheatgrass supplementation can vary with dose and experimental conditions [18,29].

None of the measured blood biochemical variables differed significantly among treatments. Most mean values were within the fish-specific ranges cited in Table 4, while ALT activity exceeded the cited reference interval in all groups. Because the elevation occurred in the control and both WGJ treatments, it cannot be attributed specifically to WGJ. Fish biochemical values are influenced by species, age, diet, rearing conditions, handling, and sampling, and broad reference intervals should not be treated as diagnostic thresholds [34,36,39,43]. The present data therefore indicate no detectable treatment-related change in the measured blood profile, but they do not establish anti-inflammatory, immunostimulatory, hepatoprotective, or renoprotective effects. Such claims would require appropriate immune markers, inflammatory mediators, histopathology, or challenge tests.

WGJ supplementation affected some components of body composition, most clearly the lower salt content in both supplemented groups. Freshwater fish regulate ion and water balance continuously, but muscle salt content alone cannot demonstrate improved osmoregulation or reduced inflammation. The biological basis of the observed difference remains uncertain because plasma ions, osmoregulatory hormones, gill transporters, and tissue water balance were not measured. The overall ANOVA for fat content was also significant, but the pairwise treatment differences should be reported only after the Tukey results have been verified. These body-composition findings warrant further study but should not be used as evidence of an anti-inflammatory effect.

The clearest treatment-related pattern was observed in the oxidative-status measurements. Activities of SOD, CAT, and GPX increased in several tissues, while MDA concentrations decreased, with the most pronounced response generally occurring at 8% WGJ. SOD converts superoxide radicals to hydrogen peroxide [53,54,55], which is subsequently removed through catalase- and glutathione-peroxidase-dependent reactions [56,57,58,59,60]; lower MDA is consistent with reduced lipid peroxidation [61,62]. Wheatgrass contains phenolic compounds, flavonoids, chlorophyll, vitamins, and other redox-active constituents that could contribute directly to radical scavenging or modulate endogenous antioxidant defenses [63,64,65,66,67]. The coordinated enzyme and MDA responses are therefore biologically consistent with enhanced antioxidant capacity. However, the study did not measure WGJ bioactive compounds in the diets, antioxidant-gene expression, or relevant signaling pathways, so the responsible mechanism cannot be established.

The culture-based microbiological results showed a different dose pattern. The 4% WGJ group had the largest descriptive reductions in total anaerobic bacteria and Enterobacteriaceae, whereas values in the 8% group were closer to those of the control. Sulfite-reducing clostridia were not detected in either supplemented group. Polyphenols and other wheatgrass constituents have antimicrobial activity and may affect bacterial growth [68,69,70], but the present study did not identify isolates to species level or measure the complete intestinal community. Total Enterobacteriaceae and sulfite-reducing clostridia are broad indicators and should not be described collectively as pathogenic bacteria. Likewise, the data do not show that Lactobacillus or Bifidobacterium increased and therefore do not establish a prebiotic effect. The non-linear pattern between 4% and 8% WGJ may reflect dose-dependent microbial sensitivity, interactions with the basal diet, or sampling variability, but these explanations remain hypotheses.

Taken together, the findings indicate that WGJ influenced physiological endpoints without producing a statistically significant improvement in growth. The stronger antioxidant response at 8% and the larger descriptive changes in selected cultivable bacterial groups at 4% also show that the response was not uniformly dose-dependent. This distinction is important for dose selection: an inclusion level that maximizes one endpoint may not maximize another. The current results support further investigation of WGJ as a candidate functional ingredient, rather than its immediate use as a replacement for antibiotics or other synthetic treatments.

The study has several limitations. Only three tanks were assigned to each treatment, the feeding period was limited, and only two WGJ inclusion levels were evaluated. The microbiological analysis was restricted to selected cultivable groups and did not characterize community composition or function. The study also did not measure immune markers, inflammatory mediators, histopathology, gene expression, WGJ bioactive-compound concentrations in the diets, or resistance to an experimental stressor or pathogen. Future studies should use longer trials, additional inclusion levels, tank-level replication, compositional analysis of WGJ-treated diets, sequencing-based microbiome methods, and targeted immune and molecular measurements.

## 5. Conclusions

The present study evaluated the effects of dietary wheatgrass juice (WGJ) supplementation at 4% and 8% inclusion levels in common carp, with particular emphasis on growth performance, blood biochemical parameters, body composition, oxidative status, and selected cultivable intestinal bacterial groups. Most growth performance parameters did not differ significantly among dietary treatments, although the viscerosomatic index was significantly lower in the WGJ-supplemented groups.

WGJ supplementation was associated with changes in several oxidative-status parameters, including increased activities of antioxidant enzymes and reduced malondialdehyde concentrations in selected tissues, with several responses being more pronounced at the 8% inclusion level. The microbiological assessment also showed changes in selected cultivable intestinal bacterial groups, particularly at the 4% WGJ inclusion level, including a reduction in sulfite-reducing clostridia.

Overall, the findings indicate that WGJ supplementation can influence selected physiological, oxidative, and cultivable intestinal bacterial parameters in common carp, although the effects were not uniformly dose-dependent and did not translate into significant improvements in most growth performance indices. Because immune, inflammatory, and broader microbial-community responses were not directly assessed, the present results should not be interpreted as evidence of immunomodulatory, anti-inflammatory, or prebiotic effects. Further studies using direct immune and inflammatory markers, comprehensive microbiome analyses, and adequately replicated tank-level experiments are warranted to clarify the mechanisms and practical relevance of WGJ supplementation in aquaculture.

## Figures and Tables

**Figure 1:**
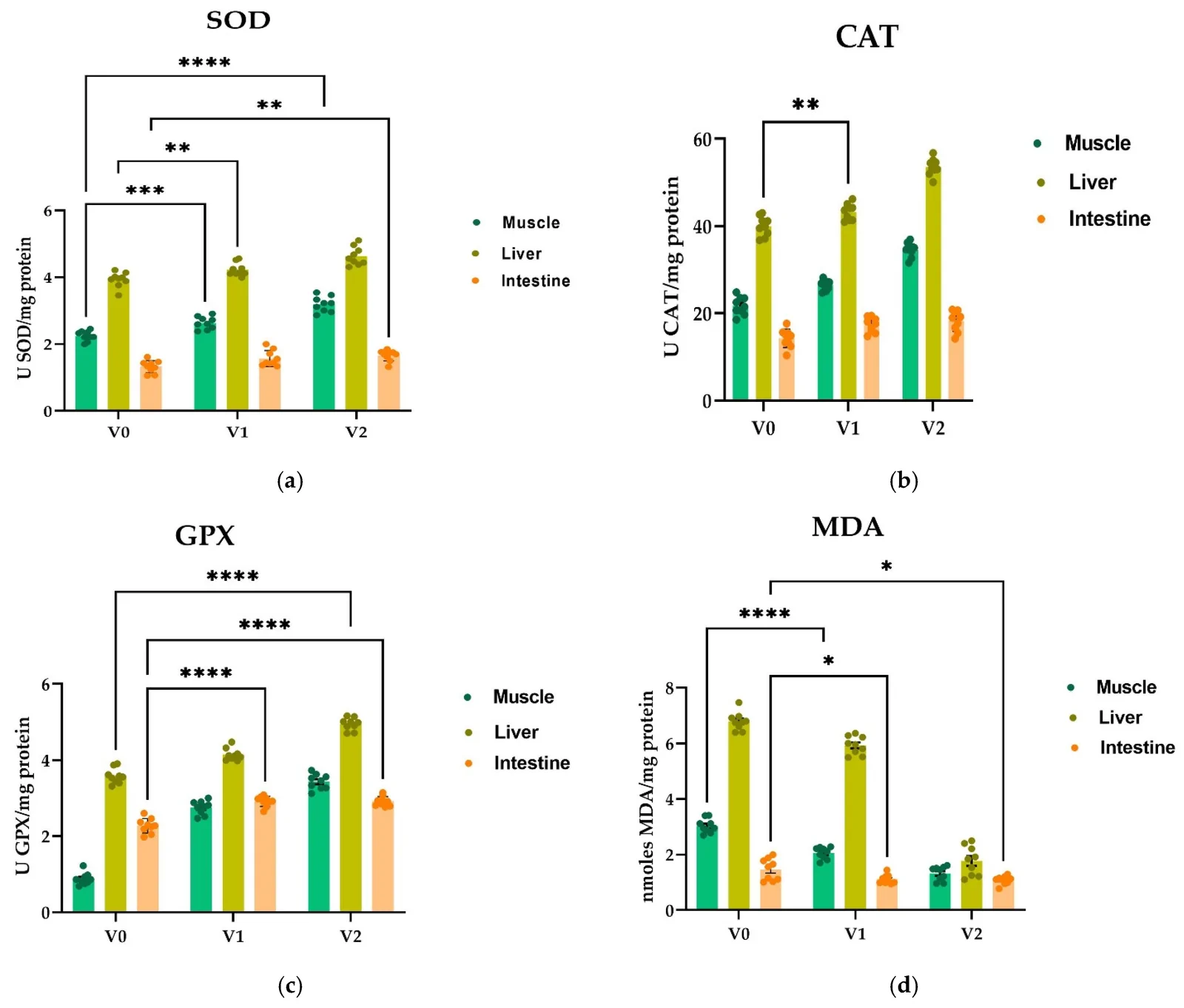
Effect of wheat juice supplementation on oxidative status in muscle, liver, and intestine of *Cyprinus carpio*. (**a**–**c**) SOD, CAT, and GPX activities; (**d**) MDA levels. Data are presented as mean ± SEM (*n* = 3). Differences among dietary groups were assessed by one-way ANOVA followed by Tukey’s test (* *p* < 0.05, ** *p* < 0.01, *** *p* < 0.001,**** *p* < 0.0001).

**Figure 2:**
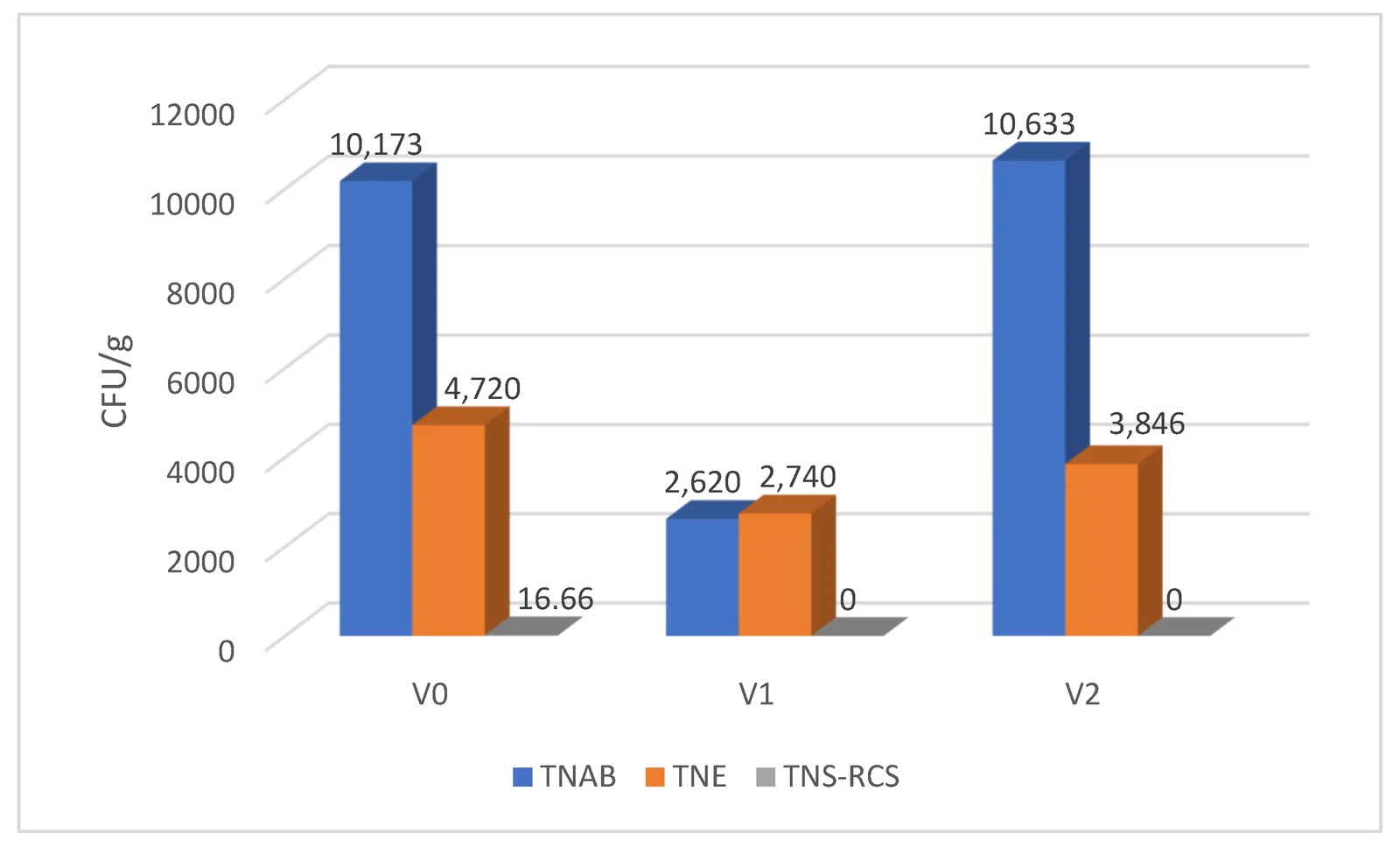
Mean values of selected cultivable intestinal bacterial indicators: total number of anaerobic bacteria (TNAB), total number of Enterobacteriaceae (TNE), and total number of sulfite-reducing clostridia (TNS-RCS), expressed as CFU/g, in common carp fed the control diet (V0), 4% WGJ diet (V1), or 8% WGJ diet (V2).

**Table 1:** Water-quality parameters in the recirculating aquaculture system during the feeding trial.

Parameter	Unit of Measurement	Value	Reference
Temperature	°C	24.8	20–25
pH	pH units	7.9	6.5–8.5
Dissolved oxygen (DO)	mg/L	7.84	>5
Nitrate (NO_3_^−^)	mg/L	10.6	<400
Nitrite (NO_2_^−^)	mg/L	0.04	<0.1
Ammonia (NH_3+_)	mg/L	0.01	<0.01
Total hardness	mg/L CaCO_3_	146	>100
Calcium (Ca^2+^)	mg/L	102	4–160
Magnesium (Mg^2+^)	mg/L	78	<15
Iron (Fe)	mg/L	0.03	<0.15
Phosphorus (P)	mg/L	0.06	3

**Table 2:** Ingredients and proximate composition of the experimental diets containing wheatgrass juice.

Ingredients (%)	V0 (0% WGJ; Control)	V1 (4% WGJ)	V2 (8% WGJ)
Fish meal	30	30	30
Meat flour	24	24	24
Sunflower meal	15	15	17
Maize	9	7	4
Wheat	9	7	4
Peas	5	5	5
DDGS	5	5	5
Soybean oil	3	3	3
WGJ	0	4	8
	Proximate composition		
Moisture	8.89	11.7	13.22
Protein	30.33	30.4	30.23
Fat	8.17	7.89	7.46
Ash	22	12.5	12.5
P	2.15	1.66	1.77
Fiber	7.49	6.38	5.02
Starch	9.8	15.6	15.6
Sugar	4.22	6.87	5.1
Ca	4.56	4.5	6.05

**Table 3:** Growth parameters of carp fed experimental diets with WGJ.

Parameter	V0 (Control)	V1	V2	*p*-Value
IBW	8.7 ± 0.13	9.07 ± 0.16	8.8 ± 0.18	0.187
FBW	28.34 ± 1.1	26.69 ± 0.71	26.89 ± 0.67	0.363
WG	17.7 ± 0.42	18.35 ± 0.72	19.62 ± 0.53	0.128
FCR	3.29 ± 0.37	3.07 ± 0.3	2.7 ± 0.14	0.359
CF	2.18 ± 0.03	2.11 ± 0.02	1.94 ± 0.15	0.156
PER	0.12 ± 0	0.12 ± 0	0.12 ± 0	0.861
RGR	0.18 ± 0.01	0.16 ± 0.01	0.17 ± 0.01	0.689
SGR	9.44 ± 0.41	9.02 ± 0.17	9.28 ± 0.39	0.693
HSI	0.24 ± 0.1	0.24 ± 0.1	0.24 ± 0.1	0.935
VSI	16.65 ± 0.36 ^a^	13.88 ± 0.84 ^b^	13.81 ± 0.74 ^b^	0.019

Different lowercase letters represent statistically significant differences according to Tukey’s test at *p* < 0.05. *p*-values were obtained by one-way ANOVA.

**Table 4:** Blood biochemical parameters of carp fed experimental diets with WGJ.

Parameter	V0 (Control)	V1	V2	*p*-Value	Normal Values	Reference
ALB (g/L)	17.33 ± 0.28	17.23 ± 0.35	17.1 ± 0.2	0.848	10–19	[35]
TP (g/L)	33.73 ± 0.49	33.43 ± 1.42	31.9 ± 0.93	0.446	28–31	[36]
GLO (g/L)	16.4 ± 0.6	16.03 ± 1.03	14.43 ± 0.77	0.271	1.4–17	[36,37]
A/G	1.07 ± 0.03	1.1 ± 0.06	1.2 ± 0.06	0.236	1.1–2.5	[38]
Ca^2+^ (mmol/L)	0.85 ± 0.37	1.14 ± 0.13	1.44 ± 0.39	0.476	1.6–1.8 ≤ 2.7	[36,39]
GLU (mmol/L)	8.82 ± 1.88	7.16 ± 1.82	7.32 ± 0.78	0.726	2.22–20.70	[40,41,42]
BUN (mmol/L)	3.45 ± 1.28	5.08 ± 0.5	5.48 ± 0.74	0.312	0.02–7.1	[36,43,44,45]
P (mmol/L)	3.05 ± 0.31	2.11 ± 0.07	2.47 ± 0.22	0.065	1.4–3.9	[46,47]
AMY (U/L)	213 ± 37.21	138.33 ± 46.45	109.67 ± 5.78	0.173	30–230	[34,48,49]
CHOL (mmol/L)	3.9 ± 0.21	3.16 ± 0.24	3.52 ± 0.42	0.299	3–12	[39,50]
ALT (U/L)	221.33 ± 29.9	150.67 ± 7.22	152.33 ± 19.47	0.091	28–124	[34,36]
ALP (U/L)	18.33 ± 4.1	21.67 ± 4.33	24 ± 3.21	0.614	11–150	[34,36,43]
CRE (µmol/L)	32 ± 3.21	35 ± 6.51	32 ± 2.65	0.863	8–45	[36]
BUN/CRE	28.67 ± 7.62	38.67 ± 7.8	43 ± 6.43	0.418	12:1/20:1	[45]
CK (U/L)	1783.33 ± 609	733 ± 163.85	1478.67 ± 360.7	0.267	270–5000	[34,36]

Values are expressed as mean ± SE (*n* = 3). *p*-values were obtained by one-way ANOVA.

**Table 5:** Proximate body composition of carp fed experimental diets with WGJ.

Parameters	Fat (%)	Humidity (%)	Protein (%)	Collagen (%)	Salt (%)
V0 (Control)	4.28 ± 0.28	77 ± 0.38	15.58 ± 0.32	1.42 ± 0.1	1.8 ± 0.15 a
V1	3.52 ± 0.2	77.38 ± 0.34	15.92 ± 0.23	1.4 ± 0.07	0.9 ± 0.14 b
V2	3.56 ± 0.15	76.84 ± 0.33	16.44 ± 0.2	1.66 ± 0.07	1.1 ± 0.19 b
*p*-value	0.048	0.547	0.095	0.072	0.05

Values are presented as mean ± SE (*n* = 3). Different lowercase letters indicate significant differences between treatments according to Tukey’s test (*p* < 0.05). ANOVA *p*-values are reported in the corresponding column.

**Table 6:** Proximate body composition of carp reported in the literature [51,52].

Parameters	Fat (%)	Humidity (%)	Protein (%)	Collagen (%)	Salt (%)
Value	8.97 ± 3.73	73.22 ± 4.32	16.6 ± 3.11	1.39 ± 0.6	1.20 ± 0.3

## Data Availability

The data supporting the findings of this study are available within the article. Further inquiries can be directed to the corresponding author.

## Abbreviations

The following abbreviations are used in this manuscript:

WGJ	Wheatgrass Juice
RAS	Recirculating Aquaculture System
IBW	Initial Body Weight
FBW	Final Body Weight
WG	Weight Gain
FCR	Feed Conversion Ratio
CF	Condition Factor
PER	Protein Efficiency Ratio
RGR	Relative Growth Rate
SGR	Specific Growth Rate
HSI	Hepatosomatic Index
VSI	Viscerosomatic Index
DO	Dissolved Oxygen
UV	Ultraviolet
DDGS	Dried Distillers Grains with Solubles
NIR	Near-Infrared
ALB	Albumin
TP	Total Protein
GLO	Globulin
A/G	Albumin/Globulin Ratio
GLU	Glucose
BUN	Blood Urea Nitrogen
AMY	Amylase
CHOL	Cholesterol
ALT	Alanine Aminotransferase
ALP	Alkaline Phosphatase
TBIL	Total Bilirubin
CRE	Creatinine
CK	Creatine Kinase
SOD	Superoxide Dismutase
CAT	Catalase
GPX	Glutathione Peroxidase
MDA	Malondialdehyde
ROS	Reactive Oxygen Species
CFU	Colony-Forming Units
TNB-A	Total Number of Anaerobic Bacteria
TNE	Total Number of Enterobacteriaceae
TNS-CSR	Total Number of Sulfite-Reducing Clostridia
NTG-A	Total Number of Aerobic Microorganisms
NT-E	Total Number of Enterobacteriaceae
NT-CSR	Total Number of Sulfite-Reducing Clostridia
PCA	Plate Count Agar
BCP	Bromocresol Purple Lactose Agar
H&E	Hematoxylin and Eosin
ANOVA	Analysis of Variance
SEM	Standard Error of the Mean
SPSS	Statistical Package for the Social Sciences
ATP	Adenosine Triphosphate (mentioned in physiological context if retained)
CO_2_	Carbon Dioxide
O_2_	Oxygen
H_2_O	Water
H_2_O_2_	Hydrogen Peroxide
CaCO_3_	Calcium Carbonate
NH_3_	Ammonia
NO_2_^−^	Nitrite
NO_3_^−^	Nitrate
Ca^2+^	Calcium Ion
Mg^2+^	Magnesium Ion
Fe	Iron
P	Phosphorus
